# The interferon-γ receptor pathway: a new way to regulate CAR T cell-solid tumor cell adhesion

**DOI:** 10.1038/s41392-022-01165-x

**Published:** 2022-09-09

**Authors:** Lingjuan Hong, Lupeng Ye

**Affiliations:** Institute of Modern Biology, Nanjing University, Nanjing, Jiangsu China

**Keywords:** Cancer therapy, Immunotherapy, Tumour immunology

A recent study published in *Nature* by the Maus group represents a great discovery that the IFNγR pathway in solid tumors affected interactions with CAR T cells by affecting cell-binding duration and avidity.^1^

Adoptive cell therapy, particularly chimeric antigen receptor (CAR) T therapy, has shown significant clinical success in leukemia, lymphoma, and multiple myeloma patients. However, CAR T cell mediates solid tumor therapy with no equivalent successes in patients. The disappointing outcomes in clinical practices can be attributed to several significant hurdles,^2,3^ such as antigen loss/down-regulation and heterogeneity of the nature of the solid tumor, CAR T cell trafficking, restricted CAR T cell infiltration and activation within tumors, poor CAR T cell expansion and persistence, T cell exhaustion. Larson et al. hypothesized that solid tumors might have more cell-intrinsic mechanisms that endow solid tumor cells to escape from CAR T cell killing in addition to the harsh solid tumor microenvironment.

**Fig. 1:**
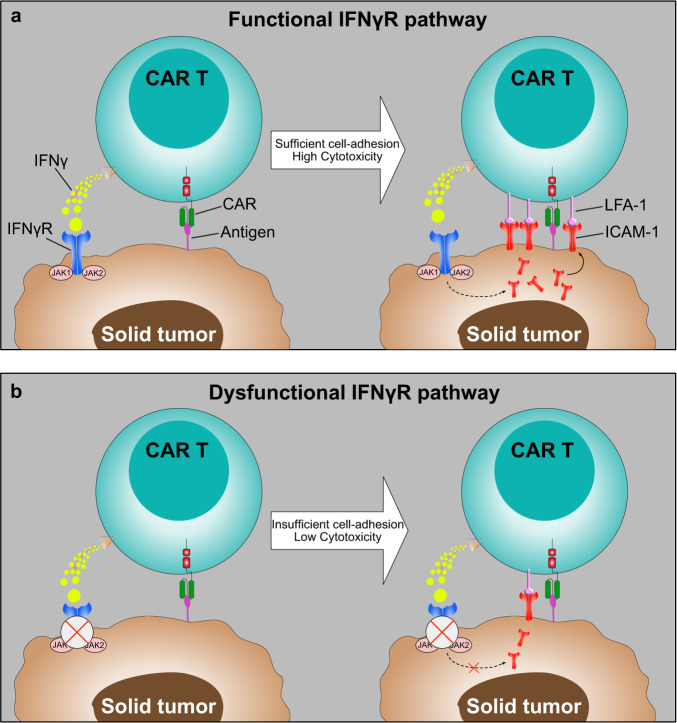
Schematics of the IFNγR signaling pathway was required for sufficient cell adhesion between CAR T and solid tumor cells. **a** A schematic of the functional IFNγR signaling pathway mediates sufficient cell adhesion by modulating CAR T cell-binding duration and avidity. High-frequency interaction between ICAM-1 and LFA-1 stabilizes the immunologic synapse, which induces effective cytotoxicity of CAR T cells against solid tumors. **b** A schematic of the dysfunctional IFNγR signaling pathway renders solid tumors more resistant to CAR T cell killing due to insufficient cell adhesion. Reduced ICAM-1 expression leads to the suboptimal immunologic synapse formation, which reduces CAR T cell killing against solid tumors

Larson et al. focused on glioblastoma, the most common and deadliest brain tumor in adults. So far, minimal clinical survival benefit has been observed in CAR T therapies and immune checkpoint blockade therapies. The authors performed a genome-scale CRISPR knockout screen using a library of more than 76,000 guide RNAs targeting 19,000 genes in human U87 glioblastoma cells. The screening assay was conducted by in vitro co-cultures of U87 cancer cells and antigen-specific EGFR-CAR T cells from several healthy donors. The MAGeCK analysis results showed that interferon-γ (IFNγ) signaling pathway-related genes, IFNγ receptor (*IFNγR1*), *IFNγR2*, and janus kinase 2 (*JAK2*), were significantly enriched. Next, the authors established U87 cells with IFNγR1, JAK1, and JAK2 knockouts, respectively. The killing assays demonstrated that these single-gene knockouts relatively rendered cancer cells resistant to CAR T cell killing. The resistance phenomenon was further validated using a subcutaneous xenograft model of U87 cells. In addition, the authors disabled IFNγR signaling using an IFNγR1 blockade, which showed significantly reduced CAR T cell killing as single-gene knockouts. To validate that resistance was independent of the CAR target, the authors tried another two models, U251 vs. IL13Rα2-CAR and U87-CD19 vs. CD19-CAR. The results consistently showed that cancer cells were relatively resistant to CAR T cytotoxicity. To determine the universality of the IFNγR pathway as a new resistance mechanism in addition to glioblastoma, authors tested other solid tumors such as pancreatic tumor cells (AsPC-1 and BxPC-3), ovarian cancer cells (SKOV3), and lung cancer cells (A549). Surprisingly, both in vitro and in vivo results exhibited that all tested solid tumor cancer cells gained more resistance to CAR T cell killing after the IFNγR pathway dysfunction Fig. 1.

Interestingly, the resistance phenomenon is not observed in hematologic malignancies, such as Nalm6 leukemic cells, Jeko-1 lymphoma, and RMPI-8226 multiple myeloma. Authors conducted expression profiling of U87 and Nalm6 tumor cells to identify the underlying mechanisms of IFNγR signaling-mediated resistance in glioblastoma but not leukemia. The differential analyses suggested that cell-adhesion pathways were significantly enriched in U87 but not Nalm6 tumor cells. Further mechanism dissection of synapses formed between CAR T cells and IFNγR1-KO U87 cells, showing lower binding avidity and shorter lifetime of formed synapses compared with wild-type U87 cells interacted with CAR T cells. The authors then examined the top differentially expressed genes between IFNγR1-KO and wild-type U87 cells and observed that Intercellular Adhesion Molecule 1 (ICAM-1) was highly expressed in wild-type but not IFNγR1-KO U87 cells after co-cultured with CAR T cells. ICAM-1 - Lymphocyte Function-associated Antigen 1 (LFA-1) is a pair of classical adhesion molecules that affect cell-cell interactions, especially in formatting immunological synapses between cancer and T cells. Notably, perturbation of ICAM-1-LFA-1 interaction by antibody-mediated blockade or knocked out ICAM-1 in U87 cells mitigated EGFR-CAR T cell killing against U87 cells, but not CD19-CAR T cells kill Nalm6 cells. In a rescue experiment, more cytotoxicity was observed in EGFR-CAR T cells when ICAM-1 was overexpressed on IFNγR1-KO cancer cells. Finally, the authors proposed a model that activated CAR T cells secreting IFNγ, which will trigger the IFNγR signaling pathway, leading to upregulation of ICAM-1 expression that will further strengthen the interaction between ICAM-1 and LFA-1 by increasing avidity and stabilizing the immunologic synapse, then increase the anti-solid tumor cell efficacy Fig. 1. This study corroborates previous findings that ICAM-1 upregulation on tumor cells is the IFNγR pathway-dependent, significantly improving CAR T cells’ entry into tumor islets.^4^ Another study published by the Maus group suggests that IFNγ is not required for CAR T cells against hematologic malignancies. However, the Han group identifies that *IFNγR1* and *IFNγR2* were significantly enriched in Nalm6 cells through a genome-wide CRISPR knockout screening;^5^ no further investigations have been performed as this paper focuses on other targets. The results imply that the loss-of-function of IFNγR1 and (or) IFNγR2 renders cancer cells resistant to CD19-CAR T cell killing differently in addition to the IFNγR pathway mediates cell adhesion which finally affects cytotoxicity.

Overall, the significance of the finding is: (1) A genome-wide CRISPR screen has been conducted in human glioblastoma and identified *IFNGR1*, *IFNGR2*, and *JAK2* that are mainly related to IFNγR signaling as top hits; (2) Loss-of-function of the IFNγR signaling pathway could specifically render glioblastoma and other solid tumors resistant phenotypes compared with wild-type cancer cells but not liquid tumors; (3) Solid tumor cell and CAR T cell-binding duration and avidity (cell adhesion) reduced due to IFNGR1 deficiency in solid tumor cells, which hurts CAR T cell cytotoxicity; (4) Liquid tumors surprisingly have been shown using different mechanisms to interact with CAR T cells, which provides critical implications for tumor-specific CAR T designs, such as enhance binding interactions between T cells and tumor cells.

The current study renders us great courage for solid tumor treatments. Optimizing CAR T cell-tumor cell adhesion alone may not be sufficient to cure solid tumors. However, if it can be combined with current strategies that have already been developed, such as immune checkpoint blockade therapy, vaccine, and oncolytic viral therapy, the synergistic effects may significantly improve the therapeutic efficacy.
